# Chronic dry eye induced corneal hypersensitivity, neuroinflammatory responses, and synaptic plasticity in the mouse trigeminal brainstem

**DOI:** 10.1186/s12974-019-1656-4

**Published:** 2019-12-17

**Authors:** Darine Fakih, Zhanlin Zhao, Pierre Nicolle, Elodie Reboussin, Fanny Joubert, Jade Luzu, Antoine Labbé, William Rostène, Christophe Baudouin, Stéphane Mélik Parsadaniantz, Annabelle Réaux-Le Goazigo

**Affiliations:** 1 Sorbonne Université, INSERM, CNRS, Institut de la Vision, 17 rue Moreau, F-75012 Paris, France; 2 R&D Department, Laboratoires Théa, 12 rue Louis Biérot, 63000 Clermont-Ferrand, France; 3 CHNO des Quinze-Vingts, INSERM-DGOS CIC 1423, 17 rue Moreau, F-75012 Paris, France; 4 Department of Ophthalmology, Ambroise Paré Hospital, AP-HP, University of Versailles Saint-Quentin-en-Yvelines, 9 avenue Charles de Gaulle, 92100 Boulogne-Billancourt, France

**Keywords:** Dry eye, Cornea, Pain, Electrophysiology, Neuroinflammation, Synaptic plasticity

## Abstract

**Background:**

Dry eye disease (DED) is a multifactorial disease associated with ocular surface inflammation, pain, and nerve abnormalities. We studied the peripheral and central neuroinflammatory responses that occur during persistent DED using molecular, cellular, behavioral, and electrophysiological approaches.

**Methods:**

A mouse model of DED was obtained by unilateral excision of the extraorbital lachrymal gland (ELG) and Harderian gland (HG) of adult female C57BL/6 mice. In vivo tests were conducted at 7, 14, and 21 days (d) after surgery. Tear production was measured by a phenol red test and corneal alterations and inflammation were assessed by fluorescein staining and in vivo confocal microscopy. Corneal nerve morphology was evaluated by nerve staining. Mechanical corneal sensitivity was monitored using von Frey filaments. Multi-unit extracellular recording of ciliary nerve fiber activity was used to monitor spontaneous corneal nerve activity. RT-qPCR and immunostaining were used to determine RNA and protein levels at d21.

**Results:**

We observed a marked reduction of tear production and the development of corneal inflammation at d7, d14, and d21 post-surgery in DED animals. Chronic DE induced a reduction of intraepithelial corneal nerve terminals. Behavioral and electrophysiological studies showed that the DED animals developed time-dependent mechanical corneal hypersensitivity accompanied by increased spontaneous ciliary nerve fiber electrical activity. Consistent with these findings, DED mice exhibited central presynaptic plasticity, demonstrated by a higher Piccolo immunoreactivity in the ipsilateral trigeminal brainstem sensory complex (TBSC). At d21 post-surgery, mRNA levels of pro-inflammatory (IL-6 and IL-1β), astrocyte (GFAP), and oxidative (iNOS2 and NOX4) markers increased significantly in the ipsilateral trigeminal ganglion (TG). This correlated with an increase in Iba1, GFAP, and ATF3 immunostaining in the ipsilateral TG of DED animals. Furthermore, pro-inflammatory cytokines (IL-6, TNFα, IL-1β, and CCL2), iNOS2, neuronal (ATF3 and FOS), and microglial (CD68 and Itgam) markers were also upregulated in the TBSC of DED animals at d21, along with increased immunoreactivity against GFAP and Iba1.

**Conclusions:**

Overall, these data highlight peripheral sensitization and neuroinflammatory responses that participate in the development and maintenance of dry eye-related pain. This model may be useful to identify new analgesic molecules to alleviate ocular pain.

## Background

Dry eye disease (DED) is a multifactorial disease of the ocular surface and tears that result in symptoms of discomfort, visual disturbance, burning, pain, and instability of the tear film [1]. Although DED is a common disorder that affects the quality of life of millions worldwide, the cellular and molecular mechanisms of the disease are not fully understood. The search for better diagnostic approaches and appropriate treatment for DED is thus the subject of intense research. Preclinical and clinical studies have shown that inflammation plays a crucial role in the pathogenesis of DED and that symptoms of pain and burning are due to neurosensory abnormalities of the corneal nerves [2–5]. It is well known that the cornea is the most densely innervated tissue in the body, and the density of nerve fibers is estimated to be 300 to 600 times that of skin [6, 7]. Sensory innervation of the cornea is provided by the nasociliary branch (containing ciliary nerves), which originates from the ophthalmic branch of the trigeminal ganglion (TG) [6, 8, 9]. Corneal neurons, which represent only 1 to 3% of the total population of trigeminal neurons, are located in the dorsomedial portion of the ophthalmic region of the TG and project central axons to the trigeminal brainstem sensory complex (TBSC) [8–10]. More precisely, the central axons of corneal sensory neurons terminate in two distinct regions of the TBSC: the trigeminal subnucleus interpolaris/caudalis (Vi/Vc) transition and the subnucleus caudalis/upper cervical cord (Vc/C1) junction regions [9, 11, 12].

Understanding the neuropathophysiological mechanisms of DED is essential for the development of new therapeutic strategies. One of the challenges of preclinical research involves the development and the characterization of relevant models that best mimic the human disease. Previous preclinical models have been developed to mimic DED using desiccative environment exposure [13, 14], the injection of anticholinergic drugs [15, 16] or botulinum toxin [17], or instillation of benzalkonium chloride [3, 18, 19]. In addition to these models, surgical aqueous tear deficiency models have also been developed in mice, consisting of excision of the extraorbital lacrimal gland (ELG) [20–23], both the extraorbital and intraorbital lacrimal glands [24], or full removal of all orbital lacrimal glands [25]. Another type of surgical model has been developed, consisting of excision of the lacrimal and Harderian glands and nictitating membrane in rabbits [26, 27]. However, the studies using these surgical models focused mainly on either the cornea or the brainstem, and none concerned the cellular and molecular mechanisms that develop along the corneal nociceptive pathways (cornea, TG, and TBSC).

In this context, we developed and characterized a preclinical mouse model of chronic DED by excision of the ELG and Harderian gland (HG) in adult female mice to affect not only stability of the tear film but also tear production, as the ELG produces the aqueous constituent of the tear film [28, 29], whereas the HG produces lipids [30]. Gland excision induced severe tear deficiency, alteration of nerve morphology, and corneal inflammation. Our results suggest that continuous DED increases spontaneous ciliary nerve fiber electrical activity, which triggers neuronal injury and the secretion of proinflammatory molecules in the TG and TBSC, thereby inducing prolonged abnormal corneal pain.

## Methods

### Experimental animals

**Fig. 1:**
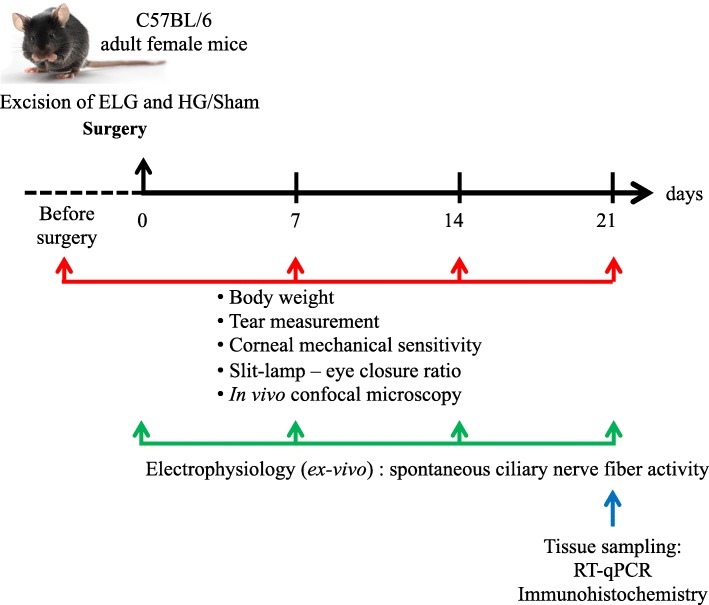
Schematic workflow of experiments for the characterization of our DED murine model. The Harderian gland (HG) and extraorbital lachrymal gland (ELG) were removed or not (sham animals). Clinical, molecular, behavioral, and electrophysiological experiments were performed from d0 to d21

Seven- to 8-week-old adult female C57BL/6 mice (average weight 18.95 ± 0.06 g) (Janvier Labs, Le Genest Saint Isle, France) were maintained under controlled conditions (22 ± 1 °C, 60 ± 10% relative humidity, 12/12-h light/dark cycle, food and water ad libitum). All animal procedures were performed in strict accordance with institutional guidelines for the care and use of experimental animals approved by the European Communities Council Directive 2010/63/UE (APAFIS #1501 2015081815454885 v2). A schematic workflow of surgical, clinical, molecular, behavioral, and electrophysiological experiments is provided in Fig. 1.

### Surgical procedures

**Fig. 2:**
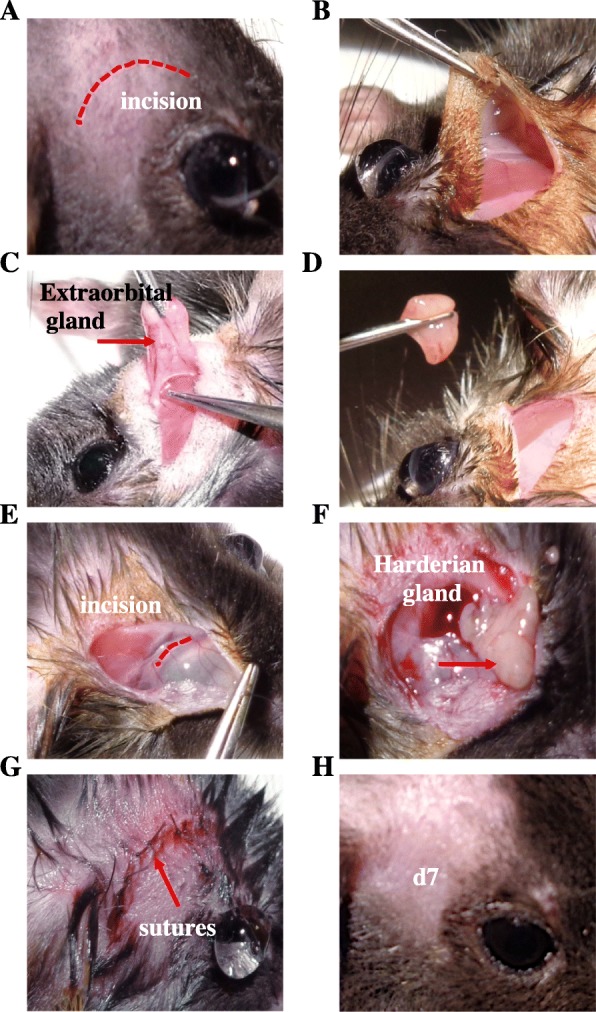
Surgical procedure for the removal of the ELG and HG. Before the surgery, a drop of lacrimal gel was applied to both eyes (**a**). Under a stereoscopic microscope, a skin incision was made (**b**) and the ELG was exposed (**c**). Next, ophthalmic forceps were used to carefully excise the ELG (**d**). An additional incision was made anteriorly towards the eye (**e**) and the HG was exposed (**e**). The incision was then sutured using 6.0 braided silk sutures (**g**). The incision was clean and showed no signs of inflammation seven days after surgery (**h**)

Unilateral (right side) ELG and HG gland excision was performed under ketamine (80 mg/kg intraperitoneal, i.p.) and xylazine (8 mg/kg i.p.) anesthesia. Before the surgery, a drop of lacrimal gel (Lubrital TM laboratory Dechra) was applied to both eyes. Under an operative microscope (Leica-Alcon II, Germany), an 8-mm skin incision on the temporal side was made to expose and remove the ELG. After dissociating the conjunctival tissue above the orbital cavity near the internal canthus, the HG was carefully removed. Complete removal was verified by inspecting the surgical area for any remaining glandular tissue. The skin incision was then sutured using 6.0 braided silk sutures (Vicryl 6-0, Ethicon, Scotland). A drop of iodine solution was applied onto the incision to avoid bacterial infection. For sham animals, an incision was made in the same zone without touching the glands. The mice were placed in warm (30 °C) cages to recover from the surgery. All steps of the surgical procedure are illustrated in Fig. 2.

### In vivo characterization of the preclinical mouse model of DED at various times after surgery

#### Tear production

Tears were measured using cotton phenol red threads (Zone-Quick, Tokyo, Japan). In a quiet room, conscious mice were taken from their cage and the top of the phenol red threads placed on the lateral canthus of the eye for 30 s. The thread is yellow in color (acidic), but changes to light red when exposed to tears. After 30 s, the length (in millimeters) of the color change was determined using a scale, as previously described. Tear production was calculated by measuring the length of thread wetting and was evaluated before in vivo confocal microscopy.

#### Eye closing ratio measurement and fluorescein staining score

Spontaneous eye closure is itself a good index for monitoring spontaneous eye pain. Spontaneous eye closure or the eye ratio is one of the quantitative measures of the grimace scale, which is used to monitor spontaneous pain behavior [31–33]. The eye closing ratio was calculated based on photographs. The eye closing ratio was calculated using the ratio height/width. The width is the distance between the internal and external canthus and height the distance between the edge of the upper eyelid and lower eyelids, going through the center of the cornea. The corneas of lightly anesthetized mice were washed with sterile 0.9% NaCl. Then a drop of fluorescein (Fluorescein Faure 0.5%) was placed into the conjunctival sac of the right eye. Ocular surface staining was then evaluated using a slit lamp under a cobalt blue light (peak of approximately 400 nm). Images were captured by a digital camera using EyeSuite™ software (Koeniz, Switzerland).

Corneal fluorescein staining was performed before and every week after the surgery to assess the degree of corneal damage caused by HG and ELG excision. The extent of corneal damage (width and intensity of fluorescein take-up area) was scored according to the following scale: 0, absence of staining; 0.5, slight punctate staining; 1, diffuse punctate staining; 2, diffuse staining covering less than one third of the cornea; 3, diffuse staining covering more than one third of the cornea; and 4, staining covering more than two thirds of the cornea [31–33].

#### In vivo confocal microscopy

An in vivo laser confocal microscope (IVCM, Heidelberg Retina Tomography (HRT) II/Rostock Cornea Module (RCM; Heidelberg Engineering GmbH, Heidelberg, Germany)) was used to examine the entire cornea of anesthetized mice [34]. The images covered an area of 400 × 400 μm with a transversal optical resolution of 2 μm and an axial optical resolution of 4 μm (Heidelberg Engineering). Approximately 100 images were acquired per animal. Image acquisitions always started in the center of the cornea at the level of the superficial epithelium and then deeper acquisitions were performed until the endothelium. Peripheral acquisitions were then performed in the same way. Experimenters were blind to the animal group.

### Animal body weight measurement

Both DED and sham animals were weighed at d0 and d7, d14, and d21 after surgery.

### RT-qPCR analysis

Anesthetized animals were perfused with sterile 0.9% NaCl. The ipsilateral TG and TBSC were quickly dissected on ice and immediately frozen using liquid nitrogen and stored at − 80 °C before use. RNA extraction from the ipsilateral TG and TBSC was performed with a NucleoSpin RNA Purification II kit (NucleoSpin RNA S, Macherey-Nagel, Hoerdt, Germany). RNA quality and concentration were then measured by the NanoDrop method (Thermo Scientific, Labtech, Uckfield, England). Reverse transcription was performed by high Capacity cDNA Reverse Transcription (Applied Biosystems, Foster City, California, USA) according to the manufacturer’s instructions. Finally, cDNA was diluted in DNAse/RNAse-free water to a final concentration of 5 ng/μL. Real-time quantitative PCR was performed with 25 ng cDNA added to 15 μL of a solution of Applied Biosystems Mastermix (TaqMan Universal PCR Master Mix) and primers for a final volume of 20 μL. The primers used were for interleukin-6 (IL-6) (Mm00446190_m1), tumor necrosis factor-α (TNFα) (Mm99999068_m1), FOS (Mm00487425_m1), ATF3 (Mm00476032_m1), chemokine (C-C motif) ligand 2 (CCL2) (Mm99999056_m1), interleukin-1β (IL-1β) (Mm00434228_m1), nitric oxide synthase 2 (iNOS2) (Mm00440502_m1), NADPH oxidase 4 (NOX4) (Mm00479246_m1), cluster of differentiation 68 (CD68) (Mm03047343_m1), glial fibrillary acidic protein (GFAP) (Mm01253033_m1), integrin alpha M (Itgam) (Mm00434455_m1), and hypoxanthine-guanine phosphoribosyltransferase (HPRT) (Mm03024075_m1). The reaction was performed on a 7300 Real-Time PCR System (Applied Biosystems). The HPRT gene was used as the endogenous reference for each reaction; mRNA levels were calculated after normalization of the results for each sample with those for HPRT mRNA. The 2^−ΔΔCt^ method was used to analyze the relative differences in specific mRNA levels between groups.

### Corneal mechanical sensitivity

Mechanical corneal sensitivity was monitored using von Frey filaments [34]. Various forces of calibrated von Frey filaments (0.008–0.04 g) were applied to the center of the cornea of immobilized mice as previously reported. The mechanical threshold corresponded to the eye-blink response. The same experimenter performed all experiments. Behavioral experiments were carried out in single-blind conditions (the experimenter was blinded to the treatment group).

### Immunohistological studies

#### Tissue preparation

Twenty-one days after surgery, anesthetized mice were transcardially perfused with 10 mL 0.9% NaCl solution followed by 40 mL 4% (w/v) paraformaldehyde in 0.1 M PBS. The eyes were carefully removed and stored at − 80 °C. The brain and TG were carefully removed and immersed in the same fixative for 24 h. The TGs were immersed in 10% (w/v) sucrose in 0.1 M PBS overnight at 4 °C and then in 30% (w/v) sucrose in 0.1 M PBS before freezing in 7.5% gelatin and 10% sucrose. Transverse frozen TGs (14 μm) and cornea (12 μm) were cut on a cryostat (Leica CM 3050 S) and mounted on Superfrost slides. Free-floating sections (40 μm) of the TBSC were prepared using a vibratome (Leica Microsystems, Germany). Sections were serially collected in 0.1 M PBS and used for immunofluorescent staining.

#### Immunofluorescence labeling

After three washes in 0.1 M PBS, TG and TBSC sections were incubated for 1 h in a blocking solution of 0.1 M PBS containing 3% normal donkey serum and 0.1% triton X-100, followed by incubation with primary antibody at 4 °C for 48 (floating brainstem sections) or 24 h (frozen TG sections). Corneal sections from naïve, sham, and DED mice were fixed in 4% PFA for 15 min, washed in 0.1 M PBS, and then blocked for 2 h at room temperature in a blocking solution of 0.1 M PBS containing 3% normal donkey serum and 0.1% triton X-100. Next, corneas were incubated overnight with primary antibody at 4 °C.

The primary antibodies used in this study were mouse anti-GFAP (Sigma-Aldrich: Lot #083 M4785, 1:250 for TGs and 1:500 for brainstem), rabbit anti-ATF3 (Santa Cruz Biotechnology: Lot #K1912, 1:250 for TG), goat anti-cFOS (Santa Cruz Biotechnology: Lot #K1715, 1:500 for brainstem), rabbit anti-Iba1 (ionized calcium-binding adapter molecule 1) (Wako: Cat. #019-19741, 1:250 for TG and 1:500 for brainstem), rabbit anti-Piccolo (1:500, ab20664 Abcam), and rabbit anti-β III tubulin (ab78078, Abcam; 1:500). Certain TG sections were incubated with fluorescein isothiocyanate-*Griffonia simplicifolia* isolectin IB4 (1:500, Vector Laboratories) overnight. All steps following incubation with the primary antibody were performed at room temperature. After three washes, ATF3, cFOS, and Piccolo staining were amplified using biotin-conjugated horse anti-rabbit antibody (1:500; Vector Laboratories) and then biotin-conjugated horse anti-goat antibody (1:500; Vector Laboratories) for 1 h and finally revealed by incubation with streptavidin-Alexa Fluor 488 (1:500; Invitrogen). Iba1 was revealed using Alexa Fluor 594-conjugated donkey anti-rabbit antibody (1:500; Invitrogen) and GFAP using Alexa Fluor 594-conjugated donkey anti-mouse antibody (1:500; Invitrogen) for 1 h. β III tubulin was revealed using Alexa 594-conjugated donkey anti-mouse antibody (Invitrogen, 1:1000). Finally, the sections were mounted onto glass slides and cover slipped.

### Microscopic analysis and immunostaining quantification

Tissue sections were examined using a Zeiss M1 epifluorescence microscope (Axio ImagerM1; Carl Zeiss). The epifluorescence microscope was equipped with a digital camera (Axio Cam HRC; Carl Zeiss) and image acquisition software (Zen; Carl Zeiss). TIFF images were obtained. The microscope was calibrated with samples from the sham mice before acquisitions of those from the DED mice. For the quantitative analysis of GFAP, Iba1, and Piccolo immunoreactivity, TG and TBSC sections were analyzed under epifluorescence microscope using a × 20 objective and the same camera parameters (Axio Vision ImagerM1; Carl Zeiss) as previously described [35]. Five ipsilateral TBSC and TG sections per animal were used for the DED and sham animals. The same gray threshold level was applied to all sections of the same series. The area within the field of interest covered by the GFAP, Iba1, and Piccolo immunoreactivity profiles relative to the total area of the measured field was measured in a completely blind manner with NIH Image J software. This value represents the percentage of the area that expressed GFAP, Iba1, and Piccolo.

### Multi-unit extracellular recording of spontaneous ciliary nerve fiber activity in ex vivo eye preparations

Spontaneous ciliary nerve fiber activity was determined at d0, d7, d14, and d21 as previously reported [34]. Briefly, mice were euthanized and the eye placed in a two-compartment chamber [34]. The cornea was continuously superfused at a rate of 3 mL/min at 33 ± 1 °C with a physiological saline solution (133.4 mM NaCl, 4.7 mM KCl, 2 mM CaCl_2_, 1.2 mM MgCl_2_, 16.3 mM NaHCO_3_, 1.3 mM NaH_2_PO_4_, and 7.8 mM glucose) saturated with O_2_ and adjusted to pH 7.4 by bubbling with 95% O_2_ and 5% CO_2_. Multi-unit extracellular electrical activity of the ciliary nerve was recorded using a suction electrode (Ag/AgCl). The signal was filtered (300–5000 Hz), amplified (× 10,000) (A-M Systems, Sequim, USA), and digitalized by Spike 2 data analysis (CED Micro1401, Cambridge Electronic Design) at a sampling frequency of 10,000 Hz. The cornea was superfused with the physiological saline solution for 30 min to stabilize the preparation before performing the electrophysiological recordings. The extracellular spontaneous ciliary nerve fiber activity was defined as impulses per second (imp/sec).

### Statistical analyses

The data obtained from sham and DED animals after surgery were compared using the appropriate paired parametric or nonparametric statistical test, as indicated. For statistical analysis, the Kolmogorov-Smirnov test was performed followed by an unpaired *t* test with Welch’s correction or nonparametric *t* test using GraphPad Prism version 7.00 (GraphPad Software, La Jolla California USA). The difference in nerve fiber impulse activity between control and injured cornea was determined using the Mann-Whitney test or unpaired *t* test as appropriate. All *P* values were considered statistically significant when the values were < 0.05. All results are presented as the mean ± standard error of the mean (SEM).

## Results

### Excision of the extraorbital lachrymal gland and Harderian gland markedly reduces tear production

**Fig. 3:**
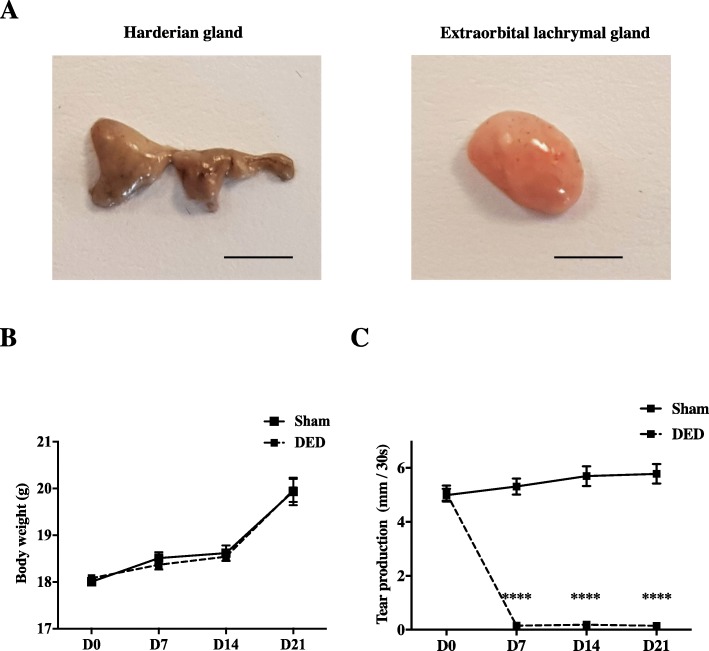
Impact of the unilateral excision of the HG and ELG on body weight and tear production. **a** Images of the right HG and ELG excised from a control mouse, scale bar = 2.5 mm and **b** sham and DED mice were analyzed for body weight. **c** Evaluation of tear production for sham and DED mice. ****P < 0.0001 relative to the sham group. Results are expressed as the mean ± SEM from d0 to d21 post-surgery. Sham animals: *n* = 60 at d0, *n* = 60 at d7, *n* = 49 at d14, and *n* = 40 at d21. DED animals: *n* = 70 at d0, *n* = 70 at d7, *n* = 58 at d14, and *n* = 42 at d21

The surgical steps for ELG and HG excision are illustrated in Fig. 2. The two glands were unilaterally excised from adult female mice (Fig. 3a). Weekly weight monitoring showed no difference between the DED and sham animals at any time (Fig. 3b). Tear production was similar before surgery between the two groups of animals (4.99 ± 0.24 mm/30 s vs 5.10 ± 0.28, *P >* 0.05) (Fig. 3c). However, there was a rapid and significant reduction in tear production by d7 (5.31 ± 0.30 vs 0.15 ± 0.04, *P* < 0.0001) (Fig. 3c), which remained significantly lower until d21 (5.70 ± 0.37 vs 0.19 ± 0.04, *P* < 0.0001, at d14, and 5.78 ± 0.36 vs 0.15 ± 0.05, *P* < 0.0001, at d21) (Fig. 3c) for the DED than sham animals.

### Excision of the extraorbital lachrymal gland and Harderian gland induces corneal epitheliopathy, reduced eye opening, and corneal inflammation

**Fig. 4:**
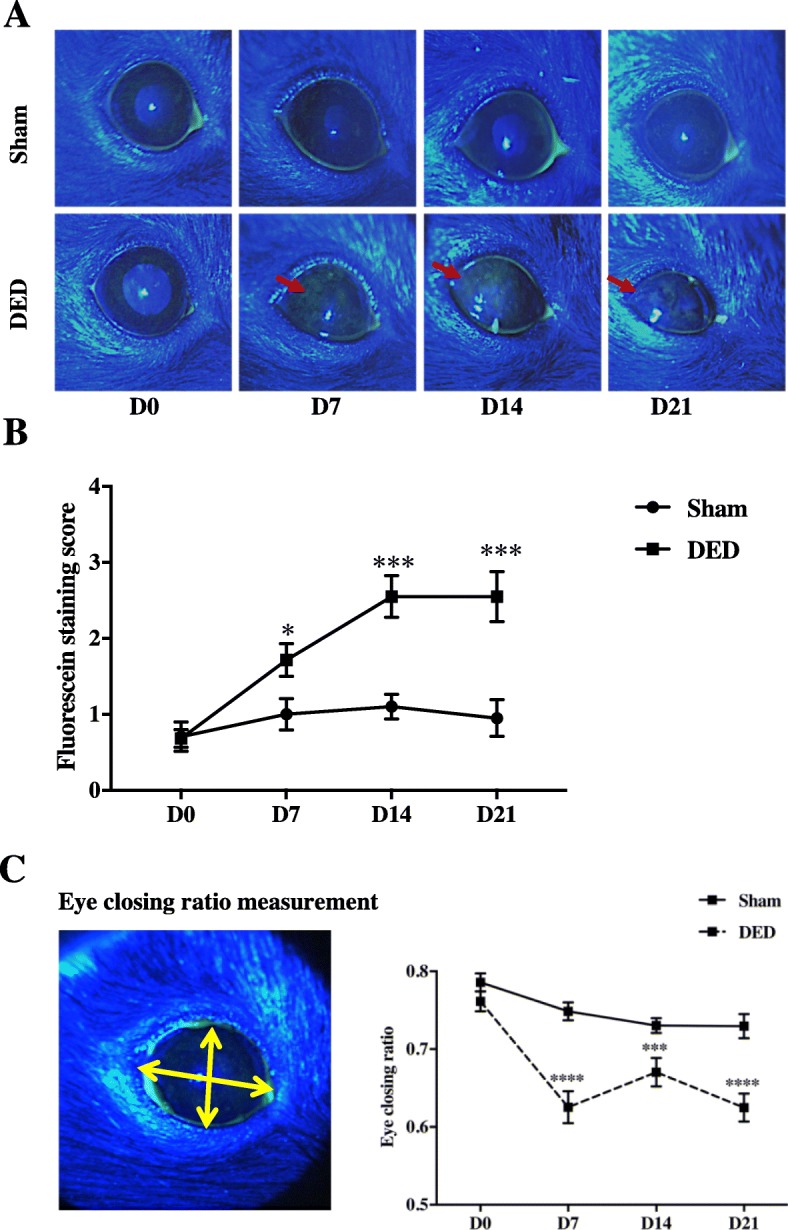
Corneal fluorescein test, fluorescein score, and eye closing ratio. **a** Cornea was examined using a slit-lamp microscope with a cobalt-blue filter after placing a drop of fluorescein (0.5% Fluorescein Faure) in the right eye of the animals. Note the punctuate keratitis at d7, d14, and d21 (red arrows). **b** Fluorescein staining was quantified at d0, d7, d14, and d21 after surgery. **c** The eye closing ratio was calculated by measuring the ratio of width/height. ***P < 0.001, ****P < 0.0001 relative to the sham group (sham animals: *n* = 34 at d0, *n* = 3 1 at d7, *n* = 27 at d14, and *n* = 28 at d21; DED animals *n* = 40 at d0, *n* = 28 at d7, *n* = 34 at d14, and *n* = 33 at d21). Results are expressed as the mean ± SEM from d0 to d21 post-surgery

We observed no difference between the two groups of animals by slit-lamp examination before surgery (d0) (Fig. 4a). Slit lamp examinations at d7, d14, and d21 showed superficial punctate keratitis (red arrows, Fig. 4a) in the cornea of the DED mice but not sham animals. The intensity of fluorescein staining was calculated before and every week after surgery using a slit-lamp ophthalmoscope and image analysis to assess corneal damage following excision of the two glands. The fluorescein scores following ELG and HG excision were significantly higher than for the sham animals at the same time point (0.70 ± 0.19 vs 0.68 ± 0.12, *P* > 0.05, at d0; 1.01 ± 0.20 vs 1.71 ± 0.21, *P* < 0.05, at d7; 1.10 ± 0.16 vs 2.55 ± 0.27, *P* < 0.001, at d14; and 0.95 ± 0.24 vs 2.55 ± 0.33, *P* < 0.001, at d21; Fig. 4b).

**Fig. 5:**
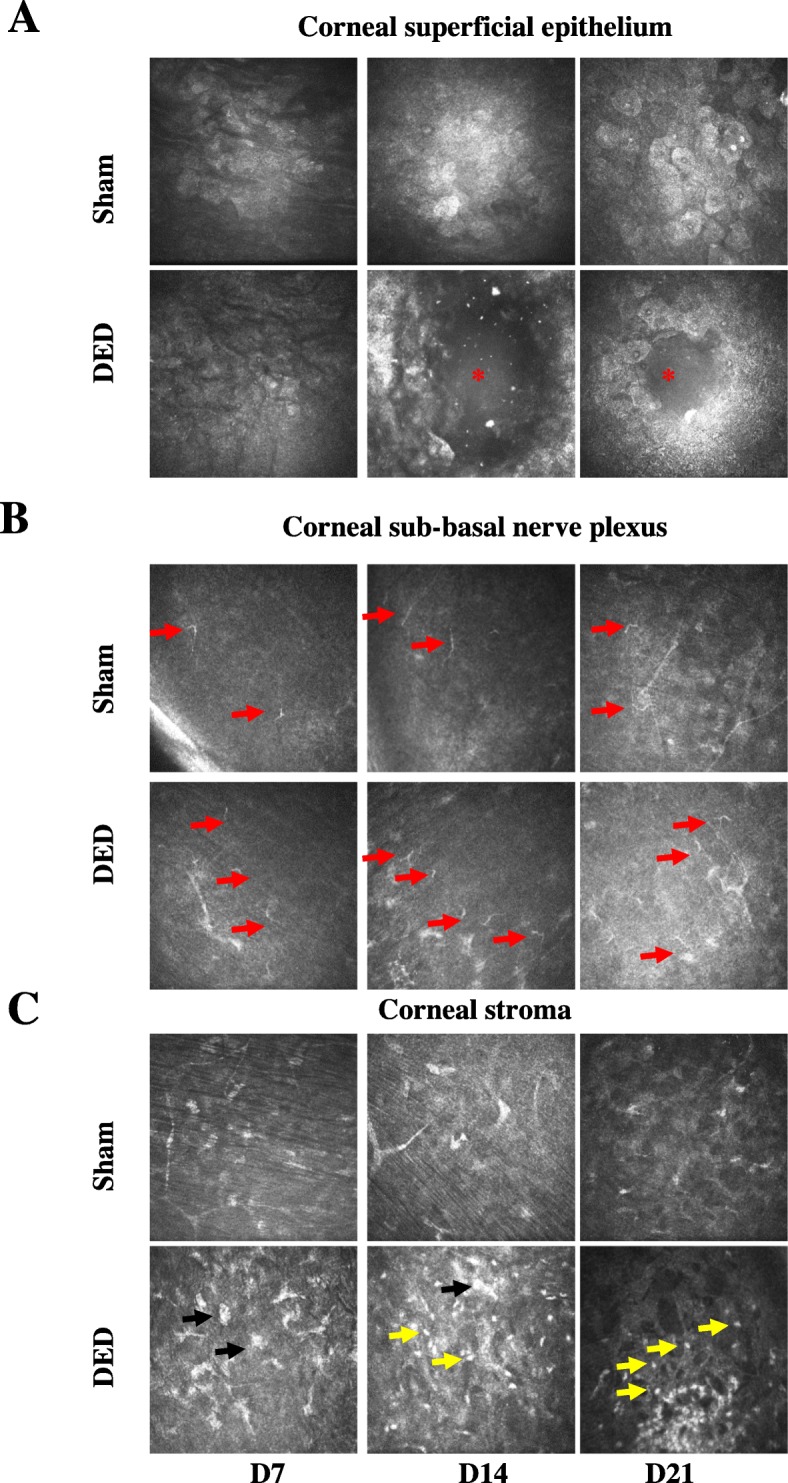
In vivo confocal microscopy images showing the different corneal layers from the sham and DED groups at d7, d14, and d21. **a** Corneal superficial epithelium (alterations of the superficial epithelium shown by red asterisks). **b** Corneal sub-basal nerve plexus (dendritic cells shown by red arrows). **c** corneal stroma (activated cells shown by black arrows and immune cells shown by yellow arrows)

We next measured the spontaneous eye closing ratio, which can be considered as an index of ocular discomfort [31–33]. There was no difference between the two groups of animals before surgery (0.78 ± 0.01 vs 0.76 ± 0.02, *P* > 0.05, at d0). However, the value for the sham animals was 0.75 ± 0.01, whereas that of the DED animals significantly dropped to 0.62 ± 0.02 (*P* < 0.0001 by d7). The spontaneous eye closing ratio remained lower in the DED animals to d21 (0.73 ± 0.01 vs 0.67 ± 0.02, *P* < 0.001, at d14, and 0.73 ± 0.02 vs 0.62 ± 0.02, *P* < 0.0001, at d21; Fig. 4c). We also monitored the corneal superficial epithelium, corneal sub-basal nerve plexus, and corneal stroma weekly by in vivo confocal microscopy. The sham animals did not exhibit damage of any of the corneal layers examined at any time point (Fig. 5). In contrast, we observed severe alterations of the superficial epithelium (d14 and d21; Fig. 5a, asterisks), long thin irregular dendritic cells in the sub-basal plexus (starting at d7; Fig. 5b, red arrows), small round hyper-reflective immune cells (Fig. 5c, yellow arrows), and activated cells in the stroma of the DED animals (Fig. 5c, black arrows).

### Extraorbital lachrymal gland and Harderian gland excision reduce the number of intraepithelial corneal nerve endings in DED animals

**Fig. 6:**
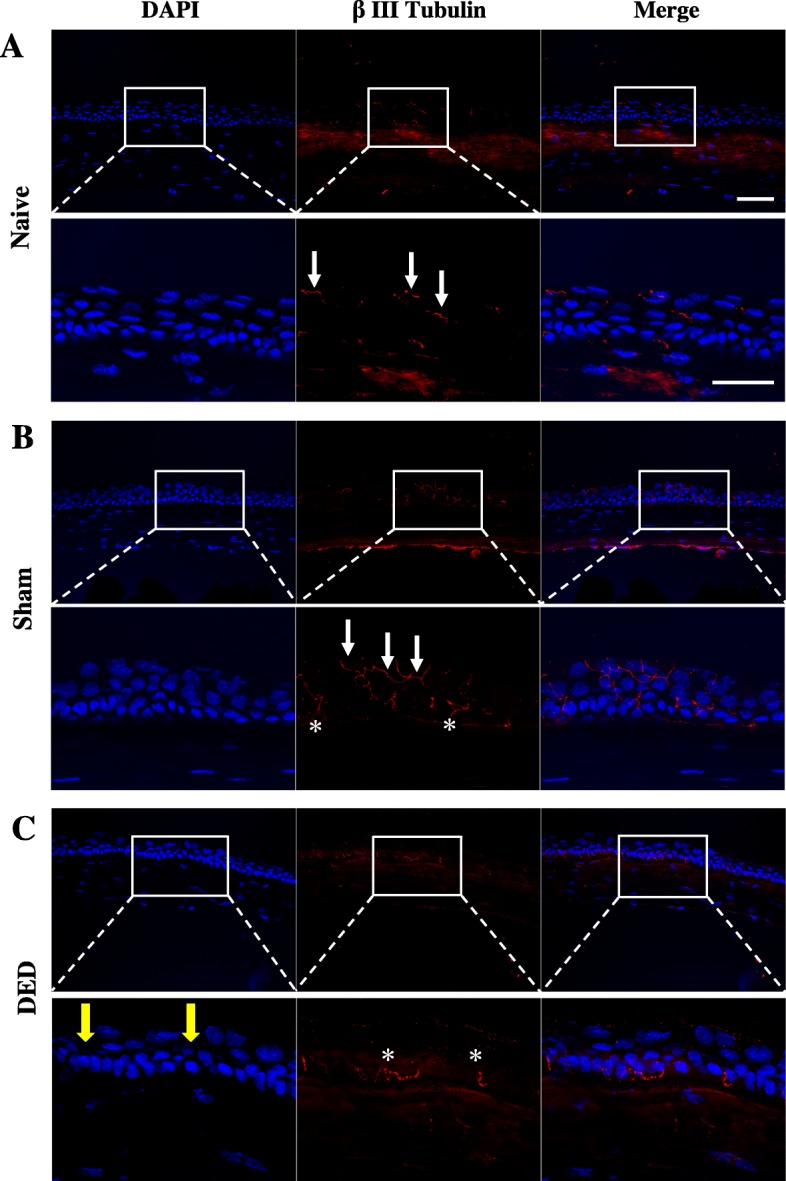
Corneal nerve immunostaining in naïve, sham, and DED mice. Confocal images (× 40 objective) from β III tubulin- and DAPI-stained cornea from naïve (**a**), sham (**b**), and DED (**c**) mice. Intraepithelial corneal nerve fibers are shown by white arrows and sub-basal nerves by white stars. Yellow arrows indicate corneal alterations induced by gland removal. The lower panel shows a × 2.8 magnification of the rectangular selection. Scale bars = 50 μm

We next assessed corneal nerve immunostaining in naïve, sham, and DED animals (Fig. 6a–c). Corneal nerves were immunostained with β tubulin antibody. DAPI staining was used to monitor corneal integrity in all animals. Corneas stained with DAPI confirmed corneal surface irregularities in DED mice relative to sham and naïve mice (Fig. 6c, yellow arrows). Low and high magnification confocal images showed clear intraepithelial corneal nerve terminals in naïve and sham animals (Fig. 6a, b; white arrows), in which the corneal axons can reach the apical surface of the cornea (white arrows). In contrast, we found fewer nerve terminals in the corneal epithelium of the DED animals (Fig. 6c). The ability of the intraepithelial axon terminals to target the apical cell layers was thus highly impaired (Fig. 6c, asterisks).

### Extraorbital lachrymal gland and Harderian gland excision induce corneal mechanical allodynia and increase spontaneous ciliary nerve fiber electrical activity

**Fig. 7:**
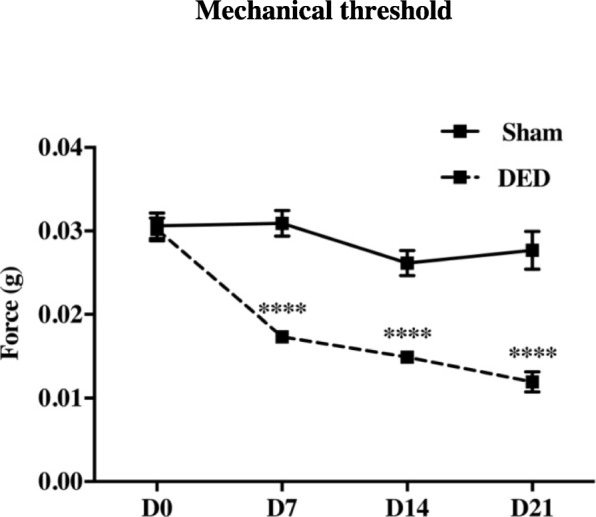
The corneal mechanical sensitivity threshold (g) of sham and DED animals was measured using von Frey filaments. ****P < 0.0001 relative to the sham group (sham animals: *n* = 38 at d0, *n* = 38 at d7, *n* = 30 at d14, and *n* = 22 at d21; DED animals *n* = 47 at d0, *n* = 47 at d7, *n* = 44 at d14, and *n* = 24 at d21). Results are expressed as the mean ± SEM from d0 to d21 post-surgery

At d0, the corneal mechanical sensitivity measured with von Frey filaments was not significantly different between the two groups (0.031 ± 0.002 g vs 0.030 ± 0.001 g, *P* > 0.05) and the corneal sensitivity remained stable over time in the sham animals (Fig. 7). In contrast, the mechanical threshold (mechanical hypersensitivity) decreased in the DED animals by d7 and was still 67% lower at d21 (0.028 ± 0.002 vs 0.012 ± 0.001 g, *P* < 0.0001; Fig. 7).

**Fig. 8:**
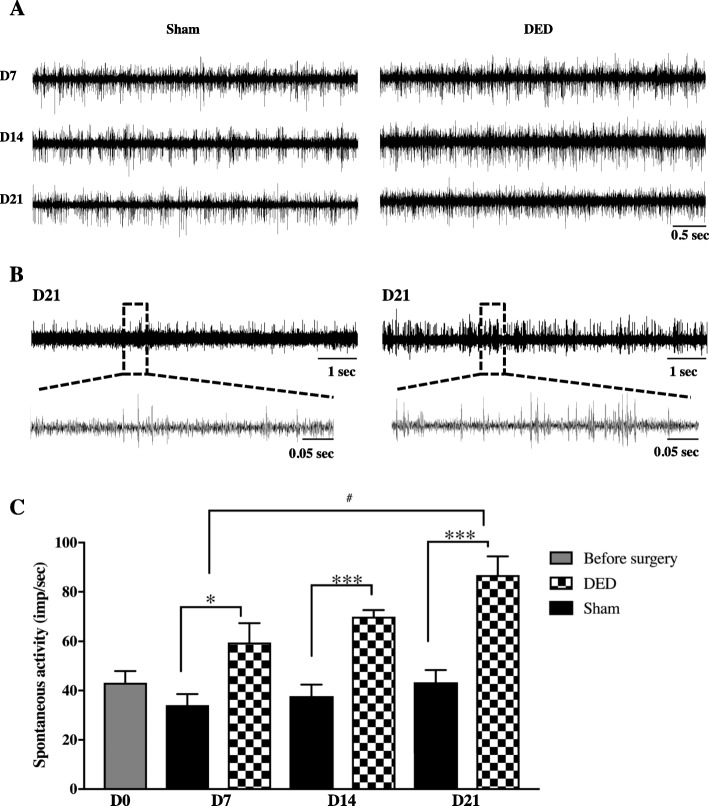
Time course of the spontaneous activity of the ciliary nerve in sham and DED mice. **a** Electrophysiological traces illustrating the extracellular activity of the ciliary nerve fiber at d7, d14, and d21 in sham and DED mice; time scale = 0.5 s. **b** Electrophysiological traces illustrating the extracellular activity of the ciliary nerve fiber at d21 in sham and DED mice; time scale = 1 s and 0.05 s. **c** Histograms showing the mean value of the spontaneous firing frequency of ciliary nerves in both groups of animals. *P < 0.05, ***P < 0.001, and ^#^*P* < 0.05 relative to the sham group (naïve animals (before surgery = d0) *n* = 6, sham animals: *n* = 5 at d7, *n* = 5 at d14, and *n* = 7 at d21; DED animals *n* = 8 at d7, *n* = 5 at d14, and *n* = 8 at d21). Results are expressed as the mean ± SEM from d7 to d21 post-surgery

We next investigated whether DED-induced corneal hypersensitivity was accompanied by an increase in the spontaneous electrical activity of the ciliary nerve at d0, d7, d14, and d21 (Fig. 8a) by extracellular recording of the ciliary nerve fiber activity [34] of naïve, sham, and DED mice. We found no difference in spontaneous ciliary nerve fiber activity between the naïve mice at d0 and sham mice at d7 (43.2 ± 4.7 vs 34.1 ± 4.5 *P* > 0.05; Fig. 8c), d14 (43.2 ± 4.7 vs 37.8 ± 4.7 *P* > 0.05; Fig. 8c), and d21 (43.2 ± 4.7 vs 43.4 ± 4.9 *P* > 0.05, Fig. 8c), demonstrating that sham surgery did not affect corneal nerve fiber activity. Quantification of the electrophysiological traces showed 74% higher basal electrical ciliary nerve activity at d7 (34.1 ± 4.5 vs 59.6 ± 7.8, *P* < 0.05; Fig. 5), 85% at d14 (37.8 ± 4.7 vs 70.1 ± 2.6, *P* < 0.001), and 100% at d21 (43.4 ± 4.9 vs 86.8 ± 7.6, *P* < 0.001; Fig. 8c) in DED than sham animals. Moreover, the spontaneous activity in DED animals at d21 was 45% higher than at d7 (86.8 ± 7.6 vs 59.5 ± 7.8, *P* < 0.05; Fig. 8c).

### Extraorbital lachrymal gland and Harderian gland excision induce neuronal injury and proinflammatory markers in the ipsilateral trigeminal ganglion.

**Fig. 9:**
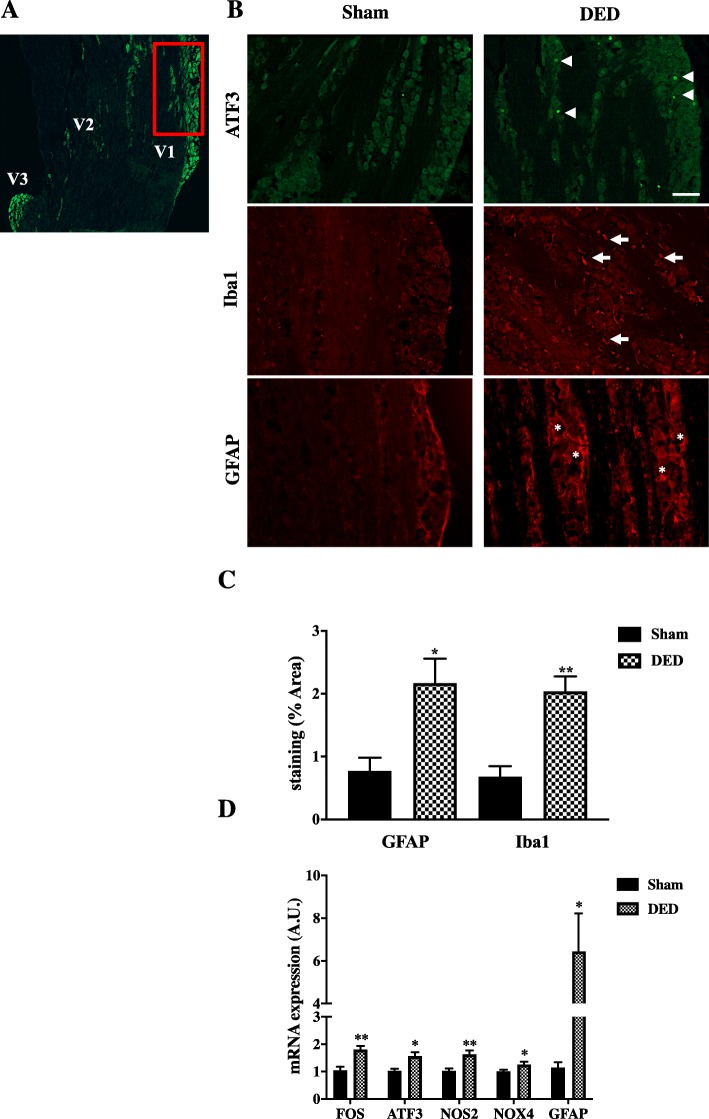
ATF3, Iba1, and GFAP immunoreactivity and expression of neuronal and inflammatory markers in the ipsilateral TG from sham and DED mice. **a** Localization of the ophthalmic branch (V1, red rectangle), maxillary branch (V2), and mandibular branch (V3) in a mouse TG using the IB4 marker. **b** ATF3 (arrowheads), Iba1 (arrows), and GFAP (asterisks) immunoreactivity of sham and DED mice at d21. Scale bar = 50 μm. **c** Quantification of Iba1 and GFAP immunoreactivity (percentage of the area immunostained with GFAP and Iba1). **d** RT-qPCR analysis of the ipsilateral TG at d21 post-surgery. *P < 0.05 and **P < 0.01 relative to the sham group (*n* = 5–9 per group). Results are expressed as the mean ± SEM at d21 post-surgery

It has been shown that satellite glial cell activation, neuronal damage, and immune cell infiltration in the TG contribute to peripheral sensitization [36]. Thus, we evaluated ATF3 (marker of neuronal damage), GFAP (satellite cells), and Iba1 (monocytes/macrophages) immunoreactivity in the ipsilateral TG d21 post-surgery. We specifically focused the microscopic evaluation at the level of the ipsilateral ophthalmic branch (V1) of the TG, (Fig. 9a, red rectangle). The persistent tear deficiency resulted in an increase in ATF3 (Fig. 9b; arrowheads), GFAP (Fig. 9b, asterisks), and Iba1-positive cells (Fig. 9b, arrows) relative to the sham animals.

We calculated the percentage of surface staining for Iba1 and GFAP for both groups (sham and DED mice). As the TG is located outside the blood-brain barrier, the infiltrating inflammatory cells labeled by the Iba1 antibody can only be derived from circulating monocytes, i.e., macrophages. This semi-quantitative analysis clearly showed significantly higher Iba1 immunoreactivity in the ipsilateral TG in the DED than the sham group (0.68 ± 0.16 vs 2.03 ± 0.24, *P* < 0.01; Fig. 9c), indicating the presence of infiltrating cells (monocytes/macrophages) following chronic DED. Quantification of GFAP immunoreactivity confirmed glial activation in the DED animals (0.77 ± 0.21 vs 2.17 ± 0.38, *P* < 0.05).

We next investigated whether such cellular changes affect gene expression in the ipsilateral TG. RT-qPCR analysis was carried out at d21. Levels of cFOS (1.80 ± 0.13-fold, *P* < 0.01), ATF3 (1.56 ± 0.14-fold, *P* < 0.01), and GFAP (6.44 ± 1.78-fold, *P* < 0.05) mRNA, as well as those of iNOS2 (1.62 ± 0.14-fold, *P* < 0.01) and NOX4 (1.32 ± 0.11-fold, *P* < 0.05) mRNA, were higher for DED than sham animals (Fig. 9d). These data clearly highlight that DED induced by excision of the ELG and the HG enhanced neuro-inflammatory responses in the TG.

### Excision of the extraorbital lachrymal gland and Harderian gland induces astrocyte and microglial activation and increased proinflammatory markers in the TBSC

**Fig. 10:**
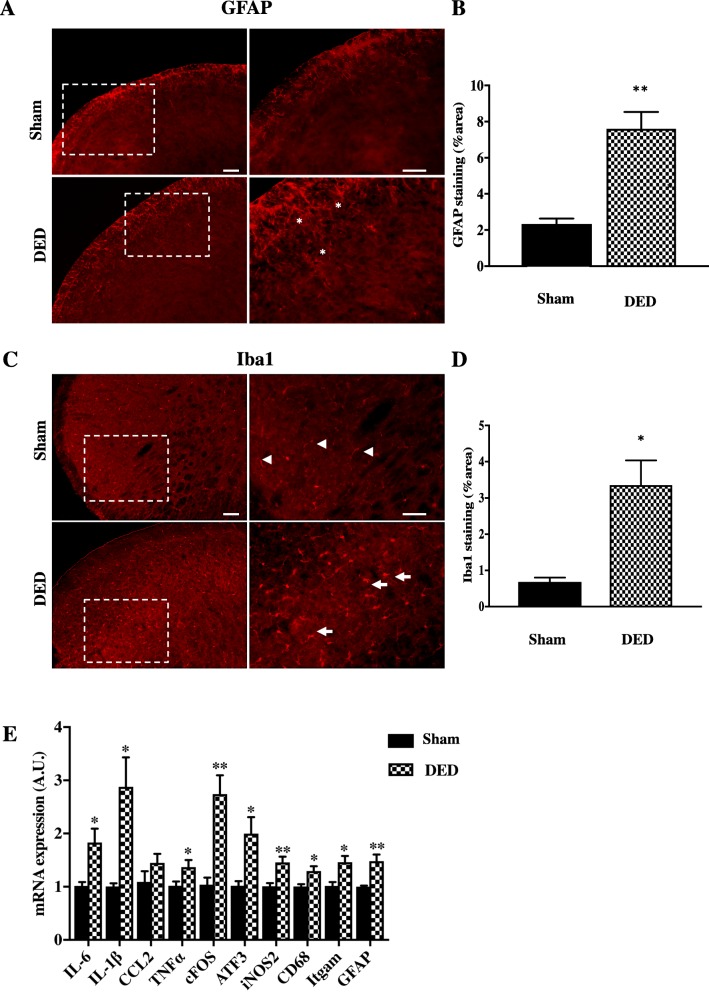
GFAP and Iba1 immunoreactivity and expression of neuronal and inflammatory markers in the ipsilateral TBSC at d21. **a** GFAP immunoreactivity from sham and DED mice. Right panels show magnification of the dashed line inset. Scale bars = 100 μm. **b** Quantification of GFAP immunoreactivity (percentage of the area immunostained with GFAP). **c** Iba1 immunoreactivity from sham and DED mice. Right panels show magnification of the dashed line inset. Scale bars = 100 μm. **d** Quantification of Iba1 immunoreactivity (percentage of the area immunostained with Iba1). **e** RT-qPCR analysis of the ipsilateral TBSC of sham and DED mice at d21 post-surgery. *P < 0.05 and **P < 0.01 relative to the sham group (*n* = 5–16 per group). Results are expressed as the mean ± SEM

It is well known that chronic pain is maintained by interactions between activated astrocytes/glial cells and microglia. We thus evaluated whether these cell populations were triggered in the TBSC 3 weeks after the surgery. We first analyzed the distribution of GFAP (Fig. 10a-b) and Iba1 (Fig. 10c, d) immunoreactivity in the ipsilateral TBSC. GFAP immunoreactivity was higher in the ipsilateral TBSC of DED mice than that of sham animals and the activated astrocytes had thickened processes with enhanced GFAP-immunoreactivity (Fig. 10a, asterisks). Semi-quantitative analysis of GFAP immunoreactivity confirmed astrocyte activation within the TBSC of DED animals relative to that of sham mice (2.33 ± 0.30 vs 7.60 ± 0.93, *P* < 0.01) (Fig. 10b). The number of Iba1-IR cells in the ipsilateral TBSC of DED mice was also higher. We detected numerous hypertrophic cell bodies (amoeboid morphological features) of activated microglia (Fig. 10c, arrows). In contrast, we observed typical resting microglia morphology (thin ramifications) in the ipsilateral TBSC of the sham mice (Fig. 10c, arrowheads). Semi-quantitative analysis clearly demonstrated higher Iba1 immunostaining in the ipsilateral TBSC of DED than sham animals (Iba1: 0.68 ± 0.12 vs 3.36 ± 0.67, *P* < 0.05; Fig. 10d).

We next examined whether molecular changes also occurred in the ipsilateral TBSC at d21 post-surgery. DED animals showed significant upregulation of neuronal activation (cFOS: 2.74 ± 0.46-fold, *P* < 0.001) and neuronal injury (ATF3: 1.20 ± 0.31-fold, *P* < 0.001) markers (Fig. 10e). Microglia and astrocytes are critical for pathological pain. Levels of the glial markers GFAP (1.48 ± 0.13-fold, *P* < 0.01), Itgam (1.46 ± 0.12-fold, *P* < 0.01), and CD68 (1.30 ± 0.09-fold, *P* < 0.05) were significantly higher in DED than sham animals (Fig. 10). The mRNA levels of the inflammatory markers IL-1β (2.88 ± 0.55-fold, *P* < 0.05), IL-6 (1.83 ± 0.30-fold, *P* < 0.05), and TNFα (1.37 ± 0.13-fold, *P* < 0.05), and the oxidative stress marker iNOS2 (1.46 ± 0.10-fold; *P* < 0.01) were also significantly higher in DED than sham animals (Fig. 10e). Such cellular changes and altered gene expression collectively show that sustained DED provoked central inflammatory responses in the ipsilateral TBSC.

### Chronic DED induces synaptic plasticity in the trigeminal brainstem sensory complex

**Fig. 11:**
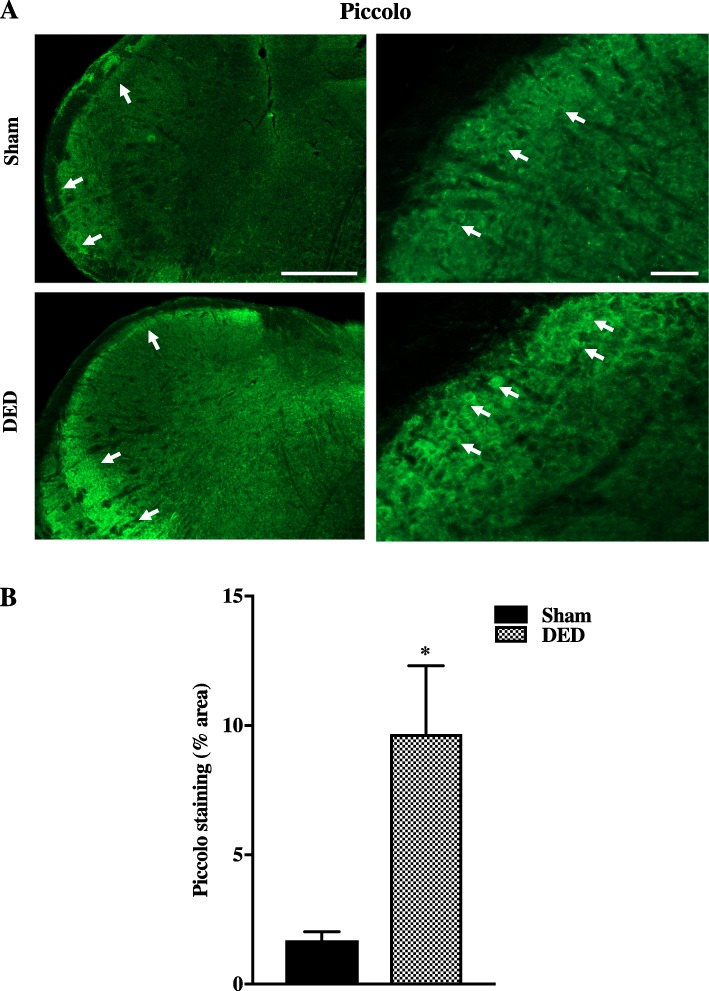
Piccolo immunostaining of the TBSC from the sham and DED groups 21 days after surgery. **a** Piccolo immunoreactivity (white arrows) from sham and DED mice. Right panels show higher magnifications. Scale bars = 500 μm for the left panel and 100 μm for the right. **b** Quantification of piccolo immunoreactivity (percentage of the area immunostained with Piccolo), *P < 0.05 relative to the sham group (*n* = 3 per group). Results are expressed as the mean ± SEM

Accumulating evidence has suggested that chronic changes of activity in primary afferent neurons induce synaptic adaptations in the central nervous system and lead to functional remodeling of presynaptic sites [37]. Piccolo is one of the components of the presynaptic zone shown to be involved in synaptic plasticity [38]. We thus investigated whether the increased spontaneous activity of the ciliary nerve fibers observed in DED mice induced changes in Piccolo immunoreactivity in the TBSC at d21. Microscopic analysis showed higher Piccolo immunostaining (white arrows) in the TBSC of DED animals than that of sham animals at low (left panels) and high magnification (right panels) (Fig. 11a). Quantification of Piccolo immunoreactivity (percentage of area immunostained by Piccolo) in the TBSC confirmed the upregulation of this presynaptic marker (1.70 ± 0.32 in sham vs 9.67 ± 2.63 in DED mice, *P* < 0.05) (Fig. 11b).

## Discussion

Dryness and pain are common debilitating symptoms of DED, affecting the quality of life of 10% of the population worldwide [39–41]. Women are more highly affected, with a Beaver Dam population-based study showing that DED affected more women than men (16.7% vs 11.4%) [42]. Reproducible and efficient DED models are needed to better understand the physiopathology of this disease and evaluate new therapeutic molecules. Here, we developed a model of DED in adult female mice obtained after the excision of the ELG and HG, as DED is divided by etiology into two categories: aqueous-deficient and evaporative disorders [1]. Our aim was to closely mimic aqueous-deficient DED by excision of the ELG and evaporative DED by excision of the HG, which produces an oily, lipid-enriched secretion.

The excision of these two functionally different glands decreased tear production by 97% by d7 post-surgery, which remained low until d21 post-surgery. This reduction of tear production is much greater than that of 30% reported after ELG and infraorbital LG excision in rats [21], 30% after ELG excision in guinea pigs [43], and 12.4% after ELG and intraorbital LG excision in the mouse [44]. This comparison highlights the efficient removal of two different functional glands (aqueous and lipid) by both markedly and rapidly reducing tear production. This result could be considered as a limitation of our model. However, we developed a model of chronic DED, as well as ocular pain, without the need to constantly inject chemical solutions or place the mice in a desiccative environment.

Ocular surface alterations and inflammation are the core mechanisms of DED [45, 46]. Here, we show that mice that underwent excision of the glands developed corneal surface alterations and inflammation by d7, which was maintained until d21 post-surgery. Among the ocular surface alterations observed, superficial punctate keratitis was first detected at d7 in DED mice, in accordance with other models of DED [16, 22, 44]. Corneal fluorescein staining demonstrated that the sham procedure did not increase corneal epitheliopathy over time, whereas severe ocular surface epitheliopathy secondary to desiccating stress was observed in the DED mice. These results are consistent with those of other preclinical DED models [17–47].

We also evaluated corneal integrity by in vivo confocal microscopy to monitor corneal inflammation, as well as changes that can occur in the different corneal layers [48, 49]. Tear deficiency induced severe alterations in the superficial corneal epithelium (desquamation) and an increase in inflammatory cells in the sub-basal plexus and corneal stroma. These in vivo results are in accordance with those of other studies performed in mice and clinical studies, which reported a decrease in superficial epithelium cell density and the presence of hyper-reflective keratocytes and inflammatory cells in the stroma [3, 26, 50, 51].

In 2017, TFOS DEWS II updated the definition of DED and added the term neurosensory abnormalities, which have been increasingly featured in the recent literature [1]. We observed that excision of HLG and ELG provoked a decrease in the mechanical corneal threshold in vivo, highlighting corneal mechanical hypersensitivity, which started at d7 and increased up to d21, suggesting changes of the corneal nerve modalities.

Electrophysiological studies show that mechanoreceptors represent 20 to 30% of corneal sensory neurons, which are the fastest conducting nociceptors stimulated by mechanical forces [6]. Such corneal hypersensitivity is consistent with that reported in the benzalkonium chloride model, corneal scraping, and corneal Alkali burns, highlighting the pain that develops under the conditions of DED [3, 34, 52]. These results are also in accordance with those of the study of Meng et al. which showed that infraorbital lachrymal gland and ELG excision in rats induced a decrease in the mechanical corneal threshold relative to that of sham rats. Of note, corneal hypersensitivity developed faster in our DED model [49].

A link between corneal hypersensitivity, inflammation, and spontaneous corneal nerve fiber activity has been previously reported in two different mouse models of corneal injury [34]. Indeed, corneal inflammation is known to participate in the activation of corneal nociceptors [53, 54], as prolonged and intense inflammation may lead to sensitization of the peripheral pathways. Such sensitization affects the modalities of nociceptors; alterations in ion channel activity and increased nerve firing and excitability have been reported [55–58]. We hypothesized that the corneal inflammation observed in DED mice may have an impact on the spontaneous activity of ciliary nerve fibers that innervate the cornea [6, 9]. We recently published an electrophysiological method for recording the global extracellular multi-unit activity of ciliary nerves in mice, allowing recording of the activity of multiple corneal nerve fibers (polymodal nociceptors, mechano-nociceptors, and cold receptors) in mice [34]. Here, we used the same experimental method to first demonstrate that there was no difference in spontaneous ciliary nerve fiber activity between naïve mice at d0 and sham mice at d7, d14, and d21, confirming that sham surgery does not affect corneal nerve fiber activity. In addition, there was a positive correlation between corneal hypersensitivity and increased spontaneous ciliary nerve fiber activity in DED animals versus sham animals from d7 until d21. This increase in spontaneous ciliary nerve fiber activity, which has been observed in another model of DED (in the guinea pig [43, 58]), became larger over the time, providing evidence for peripheral sensitization of the corneal nerves in our DED model.

Belmonte et al. elegantly showed that on-going ciliary nerve fiber activity results mostly from cold nerve fiber activity, which represents half of corneal sensory neurons [59, 60]. Thus, the increase in spontaneous ciliary nerve fiber activity observed in our mouse model may result from the activation of corneal cold nociceptors, as already reported in a preclinical model of DED performed in guinea pigs [43]. The changes in the cold nociceptor nerve modalities, which regulate tears formation and are responsible for the irritation that occurs with severe corneal dryness [61], are considered to play an etiological role in DED [43, 53, 62]. The increase of spontaneous ciliary nerve fiber activity in DED mice suggests that persistent dryness provoked corneal inflammation, which in turn affected corneal nociceptor activity, leading to the development of peripheral sensitization and corneal hypersensitivity. In addition, DED mice showed altered morphology of the corneal nerves, with a marked reduction in the number of nerve terminals at apical sites within the corneal epithelium. The reduced number of intraepithelial corneal nerve endings in DED mice 3 weeks after the surgery is consistent with previous animal [43, 63] and clinical studies [64–66]. DED mice developed corneal neuropathy in combination with altered corneal sensation (increased sensitivity) and increased ciliary nerve fiber activity. These observations are consistent with those of previous studies of Belmonte et al. [53] and Kovacs et al. [43].

Accumulating evidence suggests that chronic changes in the activity of primary afferent neurons induce synaptic adaptations in the central nervous system and lead to functional remodeling of presynaptic sites [37]. We thus more directly assessed possible presynaptic plasticity in the TBSC by evaluating the distribution of Piccolo in the region into which corneal neurons project. Piccolo, a scaffolding protein of the active zone, where synaptic vesicle fusion takes place, is considered to be a presynaptic marker. In the context of chronic pain, elevated levels of Piccolo have been reported in motor and cingulate cortical structures in a rat model of chronic neuropathic orofacial pain [37]. The authors showed that long-lasting orofacial neuropathic pain is associated with exacerbated neuronal activity and synaptic plasticity at the cortical level. Our results showing higher Piccolo immunoreactivity in the TBSC regions in chronic DED animals are in accordance with those of the aforementioned study [37]. Thus, the increase of Piccolo staining strongly indicates that profound synaptic reorganization occurs with DED. Such central reorganization, which was not yet been reported, may contribute to the chronicity of ocular pain.

Innervation of the cornea is provided by corneal neurons located in the dorso-medial part of the ophthalmic branch of the TG [8–10]. The observed increase in corneal inflammation over time led us to evaluate whether it spread to the peripheral (at the level of the TG) and central nervous system (at the level of the TBSC) by d21, when corneal alterations and spontaneous ciliary nerve fiber activity were maximal. Despite the small number of corneal neurons in the TG (only 1–3% of the total population of TG cells) [3, 67], we were able to demonstrate the upregulation of neuronal markers (FOS and activating transcription factor 3—ATF3 mRNA) in the ipsilateral TG in DED mice at d21. Such neuronal activation in the TG corroborates what we have recently reported in another model of DED induced by topical instillation of benzalkonium chloride [3]. In addition to this neuronal injury, here, we also observed increased immunoreactivity for Iba1 (monocytes/macrophages) in the ipsilateral TG of the DED animals. After peripheral nerve injury, resident macrophages are known to be activated and recruit immune cells, such as T cells and macrophages. These immune cells release several cytokines, such as IL-1, IL-6, and TNF-α [68]. TNF-α is also well known to be a proinflammatory cytokine involved in the development and maintenance of neuropathic pain. TNFα is expressed in activated Iba1-IR-positive cells, presenting morphological characteristics such as bigger cell bodies and thicker ramifications, and by activated GFAP-IR-positive cells. In addition, macrophages are believed to be the major source of TNFα in the sensory ganglion following peripheral nerve injury [69]. Furthermore, GFAP immunoreactivity was higher in the ipsilateral TG of DED animals. These activated satellite glial cells are known to express cytokines after nerve injury. Such an increase in neuronal and glial activation in the peripheral nervous system is known to produce inflammatory markers, such as IL-1β, IL-6, TNF-α, and ATF3 [70], which participate in the peripheral sensitization mechanism. This suggests that activated satellite glial cells may be another source of cytokines in the TG following DED.

Consistent with the upregulation of inflammatory markers, we observed significantly higher mRNA levels of the oxidative stress markers iNOS2 and NOX4 in the TG of DED mice than sham animals. Induced by inflammation [71, 72], iNOS2 is considered to be a signaling molecule that alters neuronal or astrocyte function [73, 74]. Increased NOX4 in the TG exerts pro-inflammatory activity by recruiting and activating monocytes/macrophages and microglia [75, 76]. Overall, the cellular and molecular changes observed in the TG after gland removal confirm the spread of corneal inflammation to the peripheral nervous system.

Several studies in preclinical models of chronic pain have shown major activation of microglia [77, 78], which are known to release numerous pro-inflammatory cytokines and chemokines, which in turn contribute to neuronal excitability and central sensitization [79, 80]. This led us to investigate whether a sustained tear deficiency induces proinflammatory responses in the TBSC. Our DED model exhibited higher Iba1 staining in the TBSC than in sham animals. Such microglial activation, which has been previously reported in the benzalkonium chloride-induced DED model [3], confirms that persistent DED has a major impact on microglial cells in the TBSC. Additionally, microglial activation was confirmed by qPCR analysis, showing a significant increase in CD68 and Itgam mRNA levels in the ipsilateral TBSC. Aside from immune cell activation, we also observed higher GFAP immunostaining in the TBSC of DED, mice suggesting that the astrocyte glial cell population participates in chronic pain.

RT-qPCR analysis revealed higher levels of mRNA for GFAP, pro-inflammatory cytokines (IL-6 and IL-1β), and oxidative stress (iNOS2) and neuronal (ATF3 and FOS) markers in the TBSC. Overall, persistent DED induced major central neuroinflammatory responses known to participate in chronic pain. Such central neuroinflammatory responses may participate in the development of chronic ocular pain observed in DED patients.

## Conclusions

**Fig. 12:**
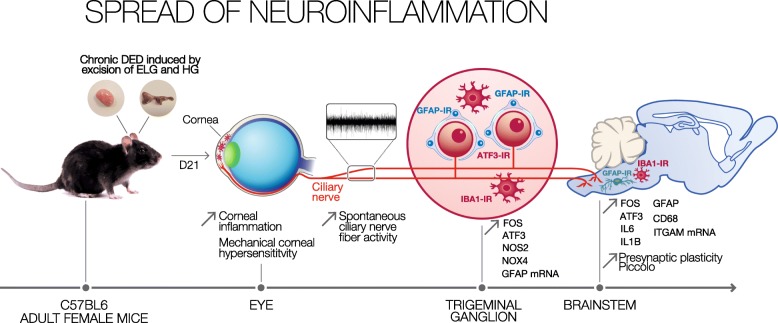
Schematic diagram showing the cellular and molecular responses induced after the excision of the ELG and HG

Here, we report the molecular, cellular, behavioral, and electrophysiological changes in the cornea, TG, and TBSC in a preclinical model of persistent DED in mice (Fig. 12). Our major observations are as follows: (1) the induction of DED in female mice by ELG and HG excision, shown by decreased tear production, increased corneal epitheliopathy, and corneal inflammation; (2) the development of spontaneous behavior consistent with ocular pain, alterations of corneal nerve morphology, and corneal mechanical hypersensitivity during the progression of DED; (3) increased spontaneous ciliary nerve fiber activity in DED animals, leading to central presynaptic plasticity in the TBSC; and (4) increased gene expression of proinflammatory, oxidative, and glial and neuronal markers in the ipsilateral TG and TBSC. These findings shed new light on how DED progresses and suggest that this mouse model of DED should be considered for preclinical testing of potential drugs for the treatment for DED and ocular pain.

## Data Availability

The datasets analyzed during the current study are available from the corresponding author upon reasonable request.

## Abbreviations

ATF3: Activating transcription factor-3
BAC: Benzalkonium chloride
CCL2: Chemokine (C-C motif) ligand 2
CD68: Cluster of differentiation 68
d: Day
DED: Dry eye disease
ELG: Extraorbital lachrymal gland
GFAP: Glial fibrillary acidic protein
HG: Harderian gland
HPRT: Hypoxanthine-guanine phosphoribosyltransferase
HRT: Heidelberg retina tomograph
Iba1: Ionized calcium-binding adapter molecule 1
IL-1β: Interleukin-1β
IL-6: Interleukin-6
iNOS2: Nitric oxide synthase 2
i.p.: Intraperitoneal
Itgam: Integrin alpha M
IVCM: *In vivo* laser confocal microscopy
NOX4: NADPH oxidase 4
RT-PCR: Real-time polymerase chain reaction
SEM: Standard error of the mean
TBSC: Trigeminal brainstem sensory complex
TG: Trigeminal ganglion
TNF-α: Tumor necrosis factor-α
Vi/Vc: Trigeminal subnucleus interpolaris/caudalis
Vc/C1: Trigeminal subnucleus caudalis/upper cervical cord
